# Species, Abundance and Function of Ammonia-oxidizing Archaea in Inland Waters across China

**DOI:** 10.1038/srep15969

**Published:** 2015-11-02

**Authors:** Leiliu Zhou, Shanyun Wang, Yuxuan Zou, Chao Xia, Guibing Zhu

**Affiliations:** 1 Key Laboratory of Drinking Water Science and Technology, Research Center for Eco-Environmental Sciences, Chinese Academy of Sciences, China; 2 Department of Biogeochemistry, Max Planck Institute for Marine Microbiology, Breme, Germany

## Abstract

Ammonia oxidation is the first step in nitrification and was thought to be performed solely by specialized bacteria. The discovery of ammonia-oxidizing archaea (AOA) changed this view. We examined the large scale and spatio-temporal occurrence, abundance and role of AOA throughout Chinese inland waters (*n* = 28). Molecular survey showed that AOA was ubiquitous in inland waters. The existence of AOA in extreme acidic, alkaline, hot, cold, eutrophic and oligotrophic environments expanded the tolerance limits of AOA, especially their known temperature tolerance to −25 °C, and substrate load to 42.04 mM. There were spatio-temporal divergences of AOA community structure in inland waters, and the diversity of AOA in inland water ecosystems was high with 34 observed species-level operational taxonomic units (OTUs; based on a 15% cutoff) distributed widely in group I.1b, I.1a, and I.1a-associated. The abundance of AOA was quite high (8.5 × 10^4^ to 8.5 × 10^9^ copies g^−1^), and AOA outnumbered ammonia-oxidizing bacteria (AOB) in the inland waters where little human activities were involved. On the whole AOB predominate the ammonia oxidation rate over AOA in inland water ecosystems, and AOA play an indispensable role in global nitrogen cycle considering that AOA occupy a broader habitat range than AOB, especially in extreme environments.

The biogeochemical nitrogen cycle is primarily driven by microorganisms with nitrification as the key process. Ammonia oxidation is the rate-limiting step of nitrification^1^,^2^ and is catalyzed by ammonia-oxidizing bacteria (AOB) and ammonia-oxidizing archaea (AOA) through ammonia monooxygenase (AMO) as the key enzyme. Studies on AOB date back to over a century ago^3^, and the knowledge about their physiology, ecology and genomics is extensive^4^,^5^,^6^,^7^. AOA were discovered recently with the identification of genes predicted to encode AMO in the phylum Crenarchaeota^8^,^9^, which aroused global scientific interest and stimulated the reassessment of the role of different ammonia oxidizers in the nitrification process.

Archaea capable of oxidizing ammonia to nitrite have been assigned to a novel phylum named Thaumarchaeota^10^,^11^, which separates them from the phyla Euryarchaeota and Crenarchaeota. They are found widely distributed in various natural environments^4^ and have considerable diversity^12^. Their physiology, ecology and genomics in marine and soil ecosystems have been widely studied^12^,^13^,^14^. However, gaps in our understanding of AOA still exist^2^,^15^,^16^. The behaviors and biogeographical distribution of AOA in inland water ecosystems are not well characterized, especially not through large-scale studies. Although the significance of AOA in ecology and microbiology has been established^17^, the specific organisms responsible for ammonia oxidation remain to be identified.

The objectives of this study are to investigate the occurrence, biodiversity and distribution of AOA in inland water ecosystems through multiple samples, including those with extreme conditions, to broaden the known tolerance limits of AOA and to examine their ecological features. This study will also test the role and influence of AOA on ammonia oxidation, and estimate their ecological potential in the aquatic nitrogen cycle. To achieve this, studies were performed in a wide range of inland water ecosystems throughout China. The abundance and community structure of AOA were analyzed using molecular methods, and the nitrification rates were measured through incubation experiments.

## Results

### Ubiquity of AOA in inland waters

**Figure 1:**
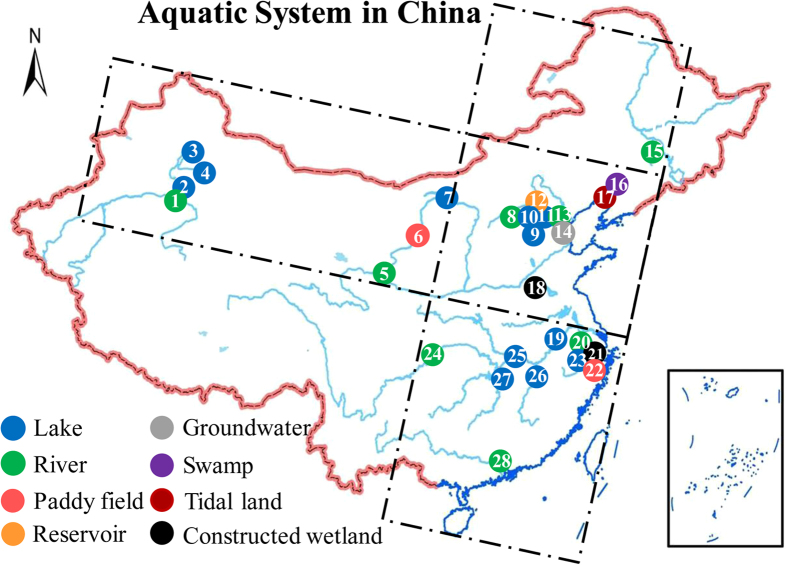
Biogeographical distribution of sampling sites in Chinese inland water ecosystems. Sites 1 to 14 were in order of longitude and sites 15 to 28 were in order of latitude as listed in Table 1. Different colors represent different types of inland waters as shown in the legend. The map were come from web of “Data Sharing Infrastructure of Earth System Science” http://www.geodata.cn. All of the maps used in the manuscript are free.

The presence of AOA in Chinese inland water ecosystems was investigated with a PCR survey of archaeal *amoA* genes resulting in the amplification of 635-bp fragments. PCR screening showed that AOA existed in all of the 100 sediment samples from the 28 sites, which verified the widespread occurrence of AOA in inland water ecosystems (Fig. 1). It is notable that AOA were still existed under extreme environmental conditions, including extremely high and low temperatures (75 °C and −25 °C), strong acidity and alkalinity (pH as low as 3.9 and up to 8.9), and oligotrophic and eutrophic conditions (Table 1).

### Community structure and diversity of AOA in inland waters

We examined the diversity of AOA and differences among AOA community structures in the inland water ecosystems. A database of 729 archaeal *amoA* gene sequences from this study was constructed and the community structures of AOA were investigated. After the diversity analysis using DOTUR software, a total of 216 unique OTUs (based on a 2% cutoff) were recovered. The observed Chao1 richness estimate and Shannon diversity index were as high as 324.90 and 4.88, indicating a high biodiversity of AOA.

**Figure 2:**
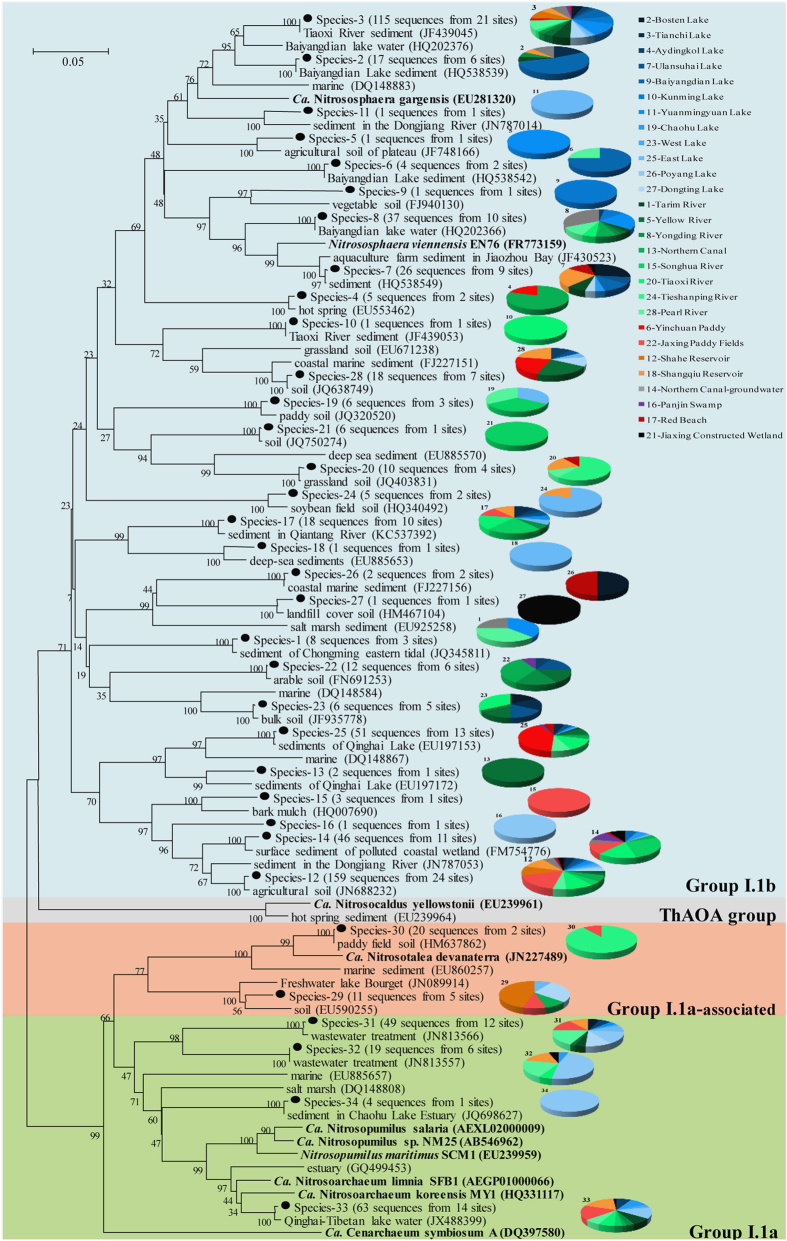
Phylogenetic tree showing the spatial divergences of AOA community structure among various inland waters. Phylogenetic trees were constructed with the neighbor-joining method using Maximum Composite Likelihood with 1000 bootstraps. The scale bar represents 5% of the sequence divergence. Pie charts for each species-level OTU show the composition of sequences from different origins with the colors corresponding to the legend.

Thirty-four species-level OTUs (each representing a specific AOA species), were obtained from our sequences, using 85% *amoA* gene sequence identity as a species threshold^18^. The evolutionary relationships between these species and published *amoA* gene sequences are shown in Fig. 2. The AOA species in Chinese inland water ecosystems were widely distributed in three AOA lineages (group I.1b, I.1a, and I.1a-associated), which are the known lineages of AOA together with the ThAOA (Thermophilic AOA) group^13^. Most of the sequences clustered into the group I.1b (28 species representing 563 sequences), while a small quantity were affiliated with the group I.1a (4 species representing 135 sequences), and the remaining two species (representing 31 sequences) were assigned to the group I.1a-associated.

Among the 34 species obtained in this study, three were found to be closely related to Candidatus *Nitrososphaera gargensis* (species-3), *Nitrososphaera viennensis* EN76 (species-7) and Ca. *Nitrosotalea devanaterra* (species-30) respectively, and one (species-33) was similar to both Ca. *Nitrosoarchaeum limnia* SFB1 and Ca. *Nitrosoarchaeum koreensis* MY1 (with identities >85.0% on the nucleotide level). This indicates that the above five known species of AOA exist in Chinese inland water ecosystems. The other 30 AOA species found in inland water sediments had not been previously characterized, which reminds us that our knowledge of AOA in natural environments is incomplete, and more unidentified AOA species probably exist^13^.

Most of our sampled inland waters harbored species-12 (24 inland waters) and species-3 (21 inland waters). 18 species existed in three or fewer of the sampled inland waters, and 11 species were specific to a particular sampling site (Fig. 2). Although some AOA species were capable of living in a broad range of environments, most of them were selective and quite a few were sensitive to different environments. The distribution of AOA species in inland water ecosystems was nonrandom on a geographical scale. As a result, the community structure of AOA varied greatly among inland waters (Supplementary Table S1). This is also a reason for the high biodiversity of AOA in inland water ecosystems.

**Figure 3:**
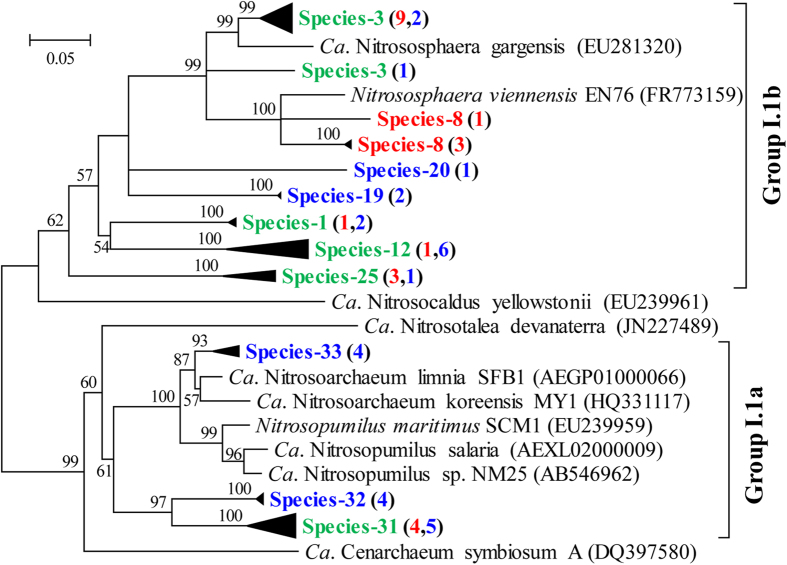
Phylogenetic tree showing the temporal divergences of AOA community structure in the Pearl River. Phylogenetic trees were constructed with the neighbor-joining method using Maximum Composite Likelihood with 1000 bootstraps. The scale bar represents 5% of the sequence divergence. Numbers in the parentheses after each species in red give the numbers of sequences obtained in summer, and blue give those in winter.

Besides the spatial distribution of AOA in Chinese inland water ecosystems, the temporal distribution of AOA was studied in the Pearl River. The AOA community structure in sediments of the Pearl River in winter and summer were analyzed (Fig. 3). Results showed that a population shift in AOA over different seasons occurred in the Pearl River, with most of the AOA in summer clustered into group I.1b, while winter AOA species distributed evenly in group I.1a and I.1b. In summary, this study documented the spatio-temporal divergences of AOA community structure.

### AOA in extreme environments

#### p*H*

Species-20 and 30 were detected in the sediments of the Tieshanping River with a pH as low as 3.9 (Table 2), indicating the strong acidity tolerance of these two species. Species-30 was identified as *Ca.* Nitrosotalea devanaterra, which has been detected in acidic soil with a pH of 4.5 and incubated under a pH of 4.0-5.5^19^. This study extended their tolerance limit to pH of 3.9. *Ca.* Nitrosotalea devanaterra (Species-30) was found to dominate the AOA population (75%) in the sediments of the Tieshanping River, with the remaining population belonging to species-20. In the soil of the Jiaxing paddy field with a pH of 6.55, *Ca.* Nitrosotalea devanaterra were also detected, accounting for a small part of the AOA population (20%). In other inland waters with a higher pH, no *Ca.* Nitrosotalea devanaterra were detected. The results provided evidence that *Ca.* Nitrosotalea devanaterra may exist only in acidic and neutral environments. The simple composition of the AOA population in the sediments of the Tieshanping River also reflected that only a few specialized AOA species could survive in environments with extreme acidity.

Eight AOA species were detected in alkaline sediments with a pH up to 8.9 (Table 2), including *Ca.* Nitrososphaera gargensis, *N. viennensis* EN76, *Ca.* Nitrosoarchaeum limnia SFB1 and *Ca.* Nitrosoarchaeum koreensis MY1, indicating their strong tolerance to alkalinity. *N. viennensis* EN76 and *Ca.* Nitrosoarchaeum koreensis MY1 were incubated under pH up to 9 and 8^20^,^21^, and *Ca.* Nitrososphaera gargensis and *Ca.* Nitrosoarchaeum limnia SFB1 were detected in environments of pH of 7.9 and 7.0–7.2^13^,^22^. This study expands their alkalinity tolerance to approximately 9. Unlike the AOA community structure in an acidic environment (Tieshanping River), that in the alkaline environment (Tarim River) was complex and spread widely between group I.1a (31.8%, 2 species) and I.1b (68.2%, 6 species).

#### Substrate

The sediment samples in this study had a wide gradient of ammonium concentrations from 0.10 mM (Tianchi Lake), to 42.04 mM (Baiyangdian Lake). Five uncharacterized AOA species belonging to group I.1b were detected in the oligotrophic Tianchi Lake (Table 2), indicating that these five species can live in environments with very low substrate concentration (0.10 mM).

*Ca.* Nitrososphaera gargensis, *N. viennensis* EN76 and four other species were observed in the sediments of Baiyangdian Lake (Table 2), indicating their strong tolerance to high ammonium concentration (up to 42.04 mM). *Ca.* Nitrososphaera gargensis was detected in environments with ammonium concentration of 5.6 mM^22^, and *N. viennensis* EN76 was incubated under ammonium concentration up to 15 mM^20^. This study expands their tolerance to high substrate content to 42.04 mM.

#### Temperature

Eight AOA species were observed in Aydingkol Lake with a high surface temperature (up to 75 °C) and six in the Songhua River with a low temperature (as low as −25 °C) (Table 2). Species-12 and 17, *Ca.* Nitrosoarchaeum limnia and *Ca.* Nitrosoarchaeum koreensis existed in both of the extreme-temperature environments, which expands their recognized temperature tolerance to between −25 °C and 75 °C. In addition, the moderately thermophilic *Ca.* Nitrososphaera gargensis, which had been detected in thermal spring microbial mats at 45 °C^22^, was also observed in Aydingkol Lake, which extends their known heat-tolerance to 75 °C. Previous studies have demonstrated the distribution of AOA in a broad range of temperature from 0.2 to 97 °C^23^,^24^,^25^. This study expands the lower limit of this range to −25 °C.

### Abundance of archaeal and bacterial ammonia oxidizers

**Figure 4:**
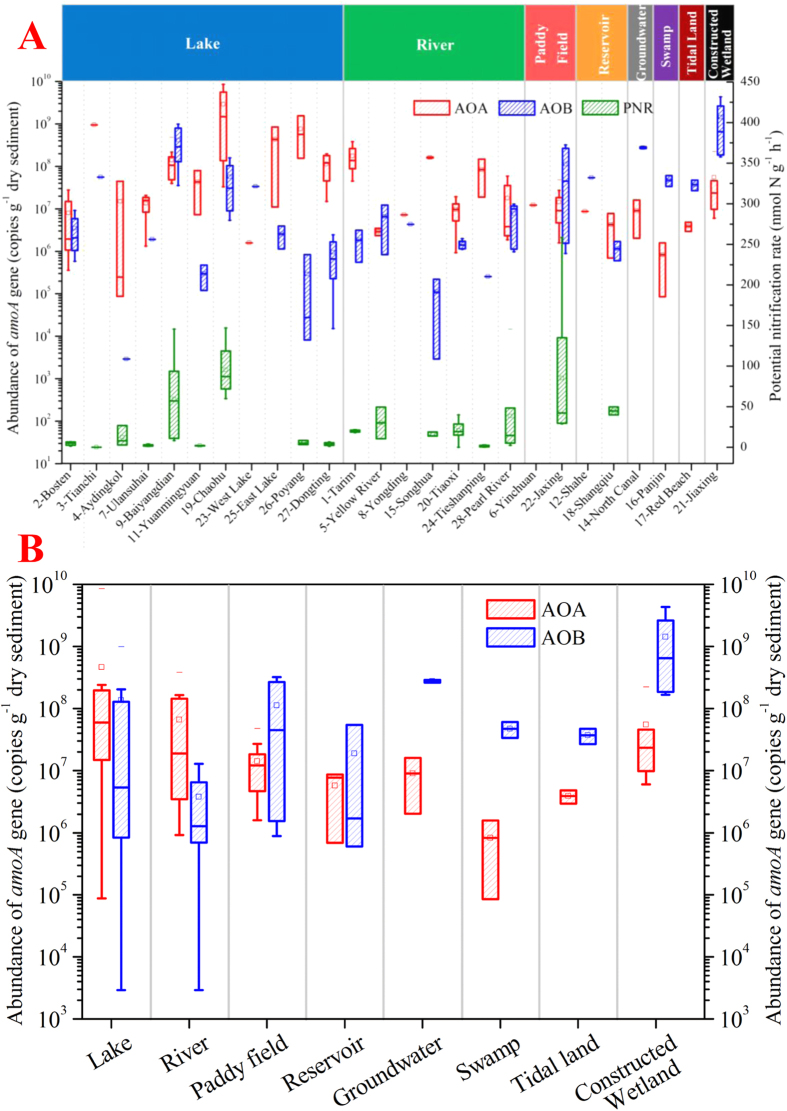
(**A**) The abundance of archaeal & bacterial *amo*A genes and potential nitrification rate (PNR) in various Chinese inland water ecosystems. (**B**) The abundance of archaeal & bacterial *amo*A genes in different types of inland waters. Boxes give the 25^th^ and 75^th^ percentiles; whiskers show the range from 1^th^ to 99^th^ percentiles; horizontal lines in and out the boxes represent the medians and maximum/minimum values respectively; little squares give the averages.

Results of qPCR showed that the abundances of AOA ranged from 8.5 × 10^4^ to 8.5 × 10^9^ copies g^−1^ dry sediment in Chinese inland waters. As the counterpart of AOA, AOB had the abundance of 2.9 × 10^3^ to 4.3 × 10^9^ copies g^−1^ (Fig. 4A). The relative abundance of AOA compared with AOB varied throughout the sampling sites (Fig. 4). and AOA outnumbered AOB in almost all the lakes and rivers, while the opposite was true in the other inland waters with strong human activities such as paddy fields, reservoirs, polluted groundwater, tidal land and constructed wetland. This result implies that AOA predominates the ammonia oxidizer population in inland waters with less human activities, while AOB dominates in inland waters with more human disturbance.

Spearman correlation analysis between archaeal & bacterial *amo*A abundance and environmental variables revealed that different parameters were related to the size of the AOA and AOB population, indicating a niche differentiation between these two groups. Archaeal *amo*A abundance had an obvious negative correlation with TC (*r* = −0.755, *p* < 0.01) and TS (*r* = −0.748, *p* < 0.01), while bacterial *amo*A abundance had an obvious positive correlation with NO_x_^–^ content (*r* = 0.497, *p* < 0.01), TN (*r* = 0.529, *p* < 0.01) and TS (*r* = 0.484, *p* < 0.01) (Supplementary Table S2).

### Contributions of AOA and AOB to microbial ammonia oxidation

Because AOA were found to be ubiquitous and abundant in Chinese inland water ecosystems, we expected that they would play a significant role in ammonia oxidation. To examine this assumption, potential nitrification rates (PNRs) were measured to estimate the combined activity of archaeal and bacterial ammonia oxidizers. The values ranged from 0 to 146.91 nmol N g^−1^ h^−1^ (*n* = 72, Fig. 4A). Spearmen correlation analysis between PNR, archaeal & bacterial *amo*A abundance and environmental variables showed bacterial *amo*A abundance and pH were significantly correlated with PNR (Supplementary Table S2). The archaeal *amo*A abundance showed no correlation with PNR. Multiple linear regression (stepwise regression) on PNR also showed that bacterial *amo*A abundance was the most determining variable for nitrification followed by pH (bacterial *amo*A abundance explained 29.5% of the variability of PNR, while pH explained 3.2%, *n* = 45, Supplementary Table S3). These results led to a possibility that AOB, rather than AOA, contribute more to nitrification in Chinese inland water ecosystems.

**Figure 5:**
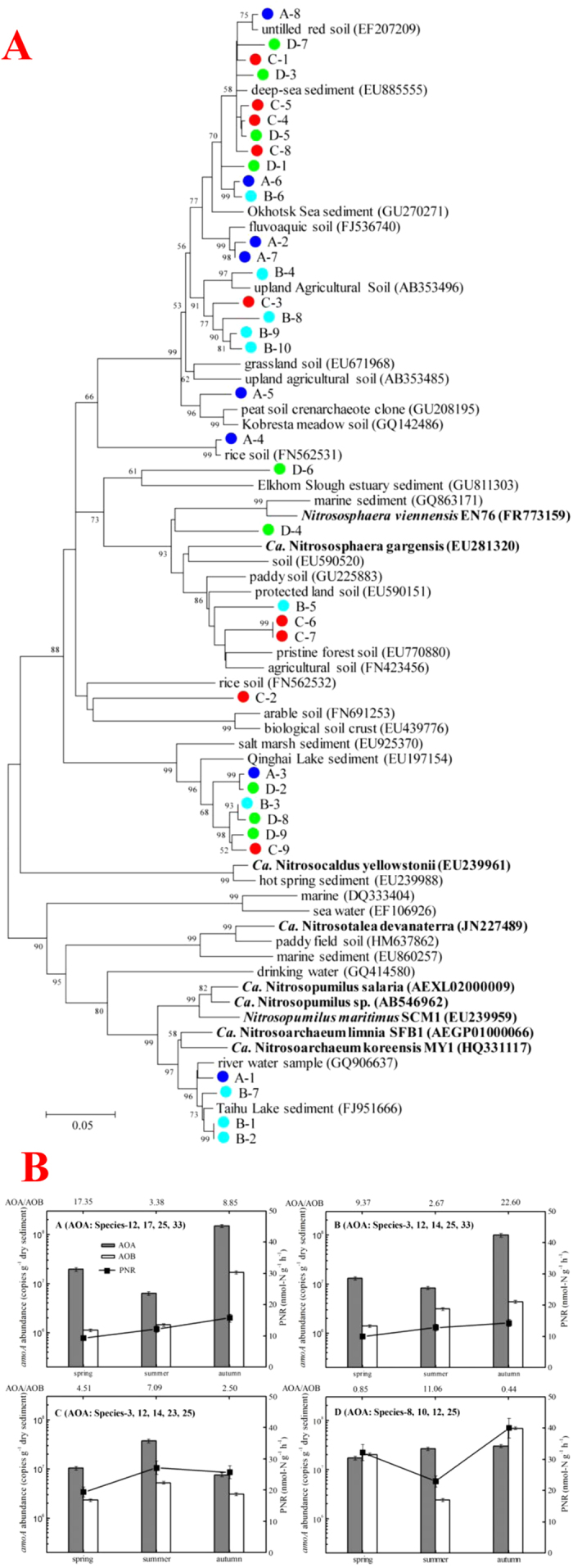
The spatio-temporal variation of AOA & AOB abundance, PNR and AOA populations in four sites on the Tiaoxi River. (**A**) Phylogenetic tree of the archaeal *amo*A gene sequences from sediments in the Tiaoxi River. The letters ABCD in the sequence names are used to distinguish sequences from different sites, marked by circles with different colors. The phylogenetic tree was constructed with the neighbor-joining method using Maximum Composite Likelihood with 1000 bootstraps. The scale bar represents 5% of the sequence divergence. (**B**)The seasonal variance in archaeal & bacterial amoA gene abundance and PNR in sediments from the Tiaoxi River. The ratios of archaeal amoA gene abundance to bacterial are listed above the diagrams. Error bars indicate standard deviation (*n* = 3).

This hypothesis was further tested in the Tiaoxi River on a spatio-temporal scale. Sediment samples from different seasons were collected from four sites in the Tiaoxi River (Supplementary Fig. S1) under different ammonia loading levels (Supplementary Table S4). The community structure of AOA (Fig. 5A) and abundance of archaeal & bacterial *amo*A genes (Fig. 5B) were detected. AOA from different sites showed quite different community structures, although species-12 and 25 existed in all of the four sites. AOA outnumbered AOB in almost all of the sediment samples, while the correlation analysis showed that the abundance of AOB not AOA had a significant correlation with PNR (Supplementary Table S5), indicating that AOB might contribute more to ammonia oxidation than AOA on spatio-temporal scale.

## Discussion

To the best of our knowledge, this is the first report of the large-scale occurrence, ecological behaviors, biodiversity and potential function of AOA in inland waters. They were found to be ubiquitous, have high biodiversity and diverse community structure, have strong adaptability to extreme conditions, abundant and play a less important role in ammonia oxidation than AOB in inland water ecosystems.

The ubiquity of AOA in inland waters was demonstrated through sediment samples (*n* = 100) from 28 inland waters, including six sites with extreme pH, temperature or substrate conditions. The ubiquity of AOA may be explained by their high diversity and strong tolerance to extreme conditions. High diversity helps increase the capacity to adapt to environmental change, and the strong tolerance to extreme conditions extends the occurrence to a largest scope^1^,^26^.

The similar phenomenon also appears in other ecosystems. AOA is also ubiquitous in soil ecosystems with quite high diversity^27^. There are few studies focused on the biodiversity of AOA in marine ecosystems on a large scale, but the observed unique archaeal *amo*A sequences specific to an individual sample location has indicated the high biodiversity of AOA in marine ecosystems^28^. For the whole natural environment, the considerable global AOA diversity was observed in a phylogenetic analysis on 12356 publicly available archaeal *amoA* sequences from different ecosystems^18^. These results demonstrate the high diversity of AOA in natural environments. As to the adaptability to extreme conditions, AOA were found in a wide pH range (2.5 to 9.0^24^,^29^), and temperature range (0.2 to 97 °C^23^,^24^,^25^), indicating the great potential of AOA in adapting to extreme acidity and alkalinity, and extreme low and high temperatures. In addition, AOA had a preference for low ammonium content^30^,^31^, and survived extreme low ammonium concentrations (≤10 nM^20^) with half-saturation constant (Km) of 133 nM total NH_4_^+^^32^. The detection of AOA in extreme conditions in this study broadened their known limits, especially those of identified species (Table 2). The cold-tolerance of AOA was expanded to −25 °C, and the tolerance to high substrate content was extended to 42.04 mM. The results point to a function of AOA in extending ammonia oxidation to a much greater range of habitats. As a result, AOA are widely distributed in marine, soil and inland water environments^1^,^13^,^33^, and exist in extreme conditions such as hot springs^34^, Antarctic waters^35^, acid soils^19^ and oligotrophic environments^32^. High AOA diversity imply a strong capacity for ammonia oxidation in various ecosystems and an important role in global nitrification.

In this study, AOA were found to numerically outcompete their counterpart AOB in inland waters with less human disturbance, while AOB dominated in inland waters affected strongly by human activities. Evidence can also be found in previous studies. AOA appear to be numerically dominant in marine environments^36^,^37^,^38^ and soils^17^,^39^,^40^,^41^, while in environments involved with more human activities like fertilized agricultural soils^42^,^43^,^44^, polluted wetlands^2^, wastewater treatment plants^45^ and bioreactors^46^,^47^, AOB outnumbered AOA. The environmental changes caused by the human activities seem to create a position of advantage for AOB over AOA. This also demonstrated the niche differentiation between AOA and AOB. The factors influencing the relative abundance of AOA to AOB were various, including the ammonia concentration^20^,^48^,^49^, the dissolved oxygen concentration^50^ and the pH^49^. Considering the complex status of various ecosystems, the relative predominance of AOA and AOB may be not affected solely by a single parameter, but by a combination of influencing factors^51^.

With the increasing understanding of AOA, the function of AOA & AOB in ammonia oxidation was called into question. This study, based on a large number of samples, documented that AOB contributed more to nitrification than AOA in Chinese inland water ecosystems. Even so, the function of AOA can’t be ignored, especially in extreme environments. In other small-scale studies, different results have been obtained. For example, nitrification was suggested be driven by bacteria rather than archaea in six nitrogen-rich grassland soils in New Zealand^30^ and in sediments of four nitrogen-rich wetlands^2^. In six estuarine sediments, PNRs increased as the abundance of archaeal *amoA* increased, rather than bacterial *amoA*^52^, and in the Black Sea water column nitrification was mainly controlled by archaeal *amoA* expression in the lower oxic zone^53^. The relative importance of AOA *vs.* AOB in ammonia oxidation needs more researches. The *in situ* measurement of microbial ammonia oxidation rate and separation of the role of AOA & AOB are two key items. In this study we can make a conclusion that AOA may play an indispensable role in global nitrogen cycle considering that AOA occupy a broader habitat range than AOB, especially in extreme environments.

## Methods

### Study site background

A total of 100 sediment samples from 28 inland water ecosystems, including lakes, rivers, paddy fields, reservoirs, groundwater, swamp, tidal land and constructed wetland were investigated in Chinese territory (23 to 46° N and 86 to 130° E). Details for every sampling site including location, background and number of samples are listed in Table 1. Other information including the nitrogenous compounds content and some physicochemical characteristics of the sampling sites is listed in Supplementary Table S6.

Among the sampled inland waters, there were six with extreme conditions. Aydingkol Lake had the highest surface temperature (75 °C) and the Songhua River had the lowest temperature (−25 °C). Tianchi Lake was an oligotrophic lake (NH_4_^+^ as low as 0.10 mM) and Baiyangdian Lake was hyper-eutrophic (NH_4_^+^ up to 42.04 mM). The sediment in Tieshanping River had an acidic pH (as low as 3.9) and Tarim River had an alkaline pH (up to 8.9). These extreme conditions were all relatively stable. Furthermore, sediments from the Pearl River and the Tiaoxi River were sampled in different seasons to verify the results on a spatio-temporal scale.

Surface sediments (0–8 cm) were collected from each sampling site in 2012 and 2013. The samples were placed in sterile plastic bags, sealed and transported to the laboratory on ice. One part of each sample was used for the analysis of physicochemical characteristics immediately after arrival, one part was incubated to measure the potential nitrification rates (PNRs), and the rest was stored at −80 °C for later DNA extraction and molecular analysis.

### DNA Extraction, PCR, Cloning and Sequences Analysis

DNA was extracted from about 0.3 g sediment using FastDNA^®^ Spin Kit for Soil (MP Biomedicals, USA). Concentrations of the extracted DNA were determined by spectrophotometric analysis on a NanoDrop 2000 UV-Vis Spectrophotometer (Thermo Fisher Scientific, USA) and the quality was checked by electrophoresis on a 1% (weight/volume percent) agarose gel. The archaeal *amoA* (ammonia monooxygenase *α*-subunit) gene was amplified using primer pairs Archaea-*amoA*F/Archaea-*amoA*R according to Francis *et al.*^28^ with an annealing temperature of 53 °C. The sequences of primers and thermal profiles used in this study were shown in Supplementary Table S7. All PCR reactions were performed with the Ex Taq^TM^ polymerase (Takara Dalian, China).

PCR amplified fragments were ligated directly using the pGEM-T^®^ Easy Vector Systems (Promega, USA) according to the manufacturer’s instructions, and then transformed to *Escherichia coli* JM109 competent cells for cloning. Selected clones were sequenced using T7 forward primers targeting vector sequences adjacent to the multiple cloning sites by an ABI PRISM 3730XL automated-sequencer (Applied Biosystems, USA). Sequences of archaeal *amoA* genes obtained in this study were deposited in the GenBank under the accession numbers (HM637849-HM637867, HQ538539-HQ538560, HQ538562, JF439021, JF439023-JF439028, JF439030-JF439044, JF439046-JF439066, KC108794-KC108815, KP167639-KP168260). All the sequences were aligned using the ClustalX 1.83 program. Phylogenetic analysis was performed using Mega 5.0 software^54^. Phylogenetic trees were constructed by neighbor-joining (NJ) with the Maximum Composite Likelihood and the robustness of tree topology was tested by bootstrap analysis with 1,000 replicates. The calculation of operational taxonomic unit (OTU) and diversity indices, including Chaol, richness estimate and Shannon diversity index, were generated by DOTUR by employing the furthest neighbor approach^55^.

### Quantitative PCR analysis

Quantitative PCR (qPCR) was performed on an ABI 7300 real-time PCR instrument (Applied Biosystems, USA) with a SYBR Green qPCR kit (Takara Dalian, China). The qPCR thermal profiles of archaeal and bacterial *amoA* genes were performed with primers Archaea-*amoA*F/Archaea-*amoA*R and *amoA-*1F/*amoA-*2R^7^, with the annealing temperatures of 53 °C and 55 °C, respectively. The detailed information on sequences of primers and thermal profiles were listed in Supplementary Table S7. Positive clones of archaeal & bacterial *amoA* gene were selected to isolate plasmids using Wizard^®^ *Plus* Minipreps DNA Purification System (Promega, USA), and standard curves were obtained with 10-fold serial dilutions of plasmid DNA containing the target genes. The results with amplification efficiency and correlation coefficient above 95% and 0.98 were employed.

### Analytical Procedures of Environmental Variables

Ammonium (NH_4_^+^), nitrate plus nitrite (NO_x_^-^) were extracted from the fresh sediment samples with 2 M KCl solution and measured using a Continuous Flow Analyzer (SAN plus, Skalar Analytical, the Netherlands). The other physicochemical characteristics (total nitrogen (TN), total carbon (TC), total sulfur (TS), total phosphorus (TP)) of the sediment samples were also measured according to Bao^56^. The pH was determined using dry sediments after mixing with water at a ratio (dry sediment/water) of 1:5, and the organic matter was determined by K_2_Cr_2_O_7_ oxidation method. All analyses were performed on triplicate samples.

### Potential nitrification rates (PNRs)

The potential nitrification rate was measured using a chlorate inhibition method with minor modifications^57^. Briefly, 3.0 g of fresh sediment was added to 50-mL centrifuge tube containing 20 mL phosphate buffer solution (NaCl 8.0 g L^−1^, KCl 0.2 g L^−1^, Na_2_HPO_4_ 0.2 g L^−1^, NaH_2_PO_4_ 0.2 g L^−1^, pH = 7.4). (NH_4_)_2_SO_4_ was added to the incubation system to a final concentration of ammonium similar to the *in situ* condition. Samples were run in triplicate with and without allylthiourea (ATU, an inhibitor of nitrification process) (100 μM final concentration) to identify the difference between NO_2_^−^ accumulation by aerobic microbial nitrification and chemical ammonia oxidation^58^. Potassium chlorate (KClO_3_) with a final concentration of 10 mg L^−1^ was added to inhibit the nitrite oxidation. The suspension was incubated (150 rpm) in the dark at *in situ* temperature for 1 h, 4 h, 8 h, 12 h and 24 h, respectively. Three subsamples were taken at each time point. The produced nitrite was extracted with 5 mL of 2 M KCl solution and determined by a spectrophotometer at wave length of 540 nm with N-(1-naphthyl) ethylenediamine dihydrochloride. The nitrification rate was calculated from the linear increase of NO_2_^–^ concentration in suspension.

### Data analysis

The statistical analyses were conducted by PASW Statistics 18.0 (Predictive Analytics Software Statistics). Mann-Whitney U test and non-parameter paired test were used respectively for the comparison of two data groups and paired data. The correlations between different types of variables were computed by Spearman correlation analysis. Stepwise linear regression analysis was used to determine the most important factor for a dependent variable. Unless otherwise specified, the level of significance in this study was α = 0.05. Graphing was achieved using Origin 8.0 software.

## Additional Information

**How to cite this article**: Zhou, L. *et al.* Species, Abundance and Function of Ammonia-oxidizing Archaea in Inland Waters across China. *Sci. Rep.* **5**, 15969; doi: 10.1038/srep15969 (2015).

## Supplementary Material

Supplementary Information

## Figures and Tables

**Table 1:** Molecular detection of AOA in Chinese inland water ecosystems.

Sample ID	Inland Waters	Locations	Background of Inland Waters			Number of samples	Number of clones sequenced	Number of species-level OTUs	Sampling season	*In situ*temperature(°C)
			Type	Sampled Environments and Backgrounds						
A: Longitudinal scale: from 86°06′E to130°10′E										
1	Tarim River	41°03′–41°04′N, 86°06′–86°07′E	Riparian zone	The longest endorheic river in China and 5th in the world		4	22	6	Summer	25
2	Bosten	41°49′–41°54′N, 86°43′–86°57′E	Lake littoral	The largest endorheic freshwater lake in China		4	20	6	Summer	20
3	Tianchi	43°54′N, 88°08′E	Lake littoral	Lake with high elevation and low temperature		1	15	5	Summer	7
4	Aydingkol	42°28′N, 89°12′E	Lake	Lowest site (–154 m) in China; 2nd in the world; highest surface temperature > 80 °C		3	19	8	Summer	75
5	Yellow River	36°05′N, 103°46′E	River	The second largest river in China		2	23	8	Summer	20
6	Yinchuan	38°16′N, 106°31′E	Paddyfield soil	Irrigated with Yellow River water		1	29	4	Summer	20
7	Ulansuhai	40°53′–40°55′N, 108°49′–108°52′E	Lake	The largest wetland on the latitude in world		4	17	8	Summer	25
8	Yongding	40°02′–40°03′N, 115°48′–115°50′E	River	The largest river in Beijing		3	11	6	Summer	22
9	Baiyangdian	38°54′–38°55′N, 115°56′–115°59′E	Ecotone	The largest lake of North China		10	33	3	Summer	24
10	Kunming	40°00′N, 116°17′E	Lake	Semi–artificial lake in the Summer Palace		1	14	3	Summer	21
11	Yuanmingyuan	40°00′N, 116°18′E	Lake	Artificial lake feeding with reclaimed water		2	35	8	Summer	23
12	Shahe	47°08′N, 116°20′E	Reservoir	Sampling from 0 cm to −40 cm with 10 cm interval		2	30	3	Summer	27
13	North Canal	40°04′N, 116°31′E	Canal	Polluted with high NH_4_^+^		6	27	7	Summer	22
14	North Canal	40°04′N, 116°31′E	Groundwater	Depth for 12 meters and 15 meters		2	34	4	Summer	12
B: Latitudinal scale: from 46°40′N to 23°08′N										
15	Songhua	44°04′–46°40′N, 125°42′–130°10′E	River riparian	The largest anabranch of Heilongjiang River		3	32	5	Winter	−25
16	Panjin	40°39–41°27′N, 121°25–122°31′E	Swamp	One of the wetlands preserved best in the world		2	10	4	Summer	22
17	Red Beach	40°41′–41°27′N, 121°31′–122°28′E	Tidal land	Polluted seawater		4	12	6	Summer	24
18	Shangqiu	34°38′N, 115°58′E	Reservoir	On the second largest river of China		2	58	9	Summer	26
19	Chaohu	31°33′–31°41′N, 117°24′–117°47′E	Lake	The fifth largest freshwater lake in China		4	14	4	Autumn	20
20	Tiaoxi	30°11′–30°15′N, 119°37′–119°44′E	River	The largest tributary of Taihu lake, the third largest lake of China		5	44	9	Summer	24
21	Jiaxing	30°46′–30°47′N, 120°42′–120°43′E	Constructed wetland	The largest constructed wetlands in China		6	12	5	Summer	22
22	Jiaxing	30°46′N, 120°42′E	Paddyfield	Sampling from surface to −1 m with 10 cm interval		10	59	7	Summer	20–24
23	West Lake	30°15′N, 120°09′E	Lake	Famous scenery in Hangzhou Province		1	9	5	Summer	26
24	Tieshanping	29°36′–29°37′N 106°40′–106°41′E	River	Catchment area in the watershed was polluted by acid rain		2	24	2	Summer	28
25	Donghu	30°34′N, 114°23′E	Lake littoral	The largest city lake of China		3	21	11	Late spring	19
26	Poyang	29°24′–29°26′N, 116°01′–116°02′E	Lake	The largest freshwater lake in China		3	33	6	Summer	21
27	Dongting	29°20′–29°22′N, 113°05′–113°06′E	Lake littoral	The second largest freshwater lake in China		4	22	5	Summer	23
28	Pearl River	23°08′–23°09′N, 113°10′–113°11’E	Estuary	The third largest river in China	Winter (PRW)	3	28	9	Winter	13
					Summer (PRS)	3	22	6	Summer	28

**Table 2:** AOA species in extreme environments of Chinese inland waters. Species numbers were in accordance with those in Fig. 2.

	Extreme Condition	Inland Waters	AOA Species	Known Species	Original Limits	
					Natural environments	Incubated environments
pH	as low as 3.9	Tieshanping River	species-20, 30	*Ca.* Nitrosotalea devanaterra	4.5^19^	4.0–5.5^19^
	up to 8.9	Tarim River	species-2, 3, 7, 8, 12, 25, 31, 33	*Ca.* Nitrososphaera gargensis	7.9^22^	7.4^59^
				*N. viennensis*	8^20^	5–9^20^
				*Ca.* Nitrosoarchaeum limnia	7.0–7.2^13^	—
				*Ca.* Nitrosoarchaeum koreensis	5.6^21^	6–8^21^
Substrate	as low as 0.10 mM	Tianchi Lake	species-2, 12, 17, 23, 25	None	—	—
	up to 42.04 mM	Baiyangdian Lake	species-2, 3, 6, 7, 12, 14	*Ca.* Nitrososphaera gargensis	5.6 mM^22^	0.14–3.08 mM^59^
				*N. viennensis*	—	1–15 mM^20^
Temperature	75 °C	Aydingkol Lake	species-3, 8, 12, 17, 22, 28, 31, 33	*Ca.* Nitrososphaera gargensis	45 °C^22^	46 °C^59^
				*Ca.* Nitrosoarchaeum limnia	21.6 °C^60^	—
				*Ca.* Nitrosoarchaeum koreensis	—	15–30 °C^21^
	−25 °C	Songhua River	species-12, 14, 17, 19, 21, 33	*Ca.* Nitrosoarchaeum limnia	21.6 °C^60^	—
				*Ca.* Nitrosoarchaeum koreensis	—	15–30 °C^21^
