# Supplementary material for: Species, Abundance and Function of Ammonia-oxidizing Archaea in Inland Waters across China
Source: Sci Rep. 2015 Nov 2;5:15969. doi: 10.1038/srep15969 (PMC4629152; doi:10.1038/srep15969)
Supplement: Supplementary Information [file srep15969-s1.pdf]

Species, Abundance and Function of Ammonia-oxidizing Archaea in Inland Waters across China

Leiliu Zhou[1], Shanyun Wang[1], Yuxuan Zou[1], Chao Xia[1], Guibing Zhu[1*]

1. Key Laboratory of Drinking Water Science and Technology, Research Center for Eco-Environmental Sciences, Chinese Academy of Sciences, Beijing, China

Supporting Information containing 4 pages, with 1 figure and 7 tables.

Supporting Information

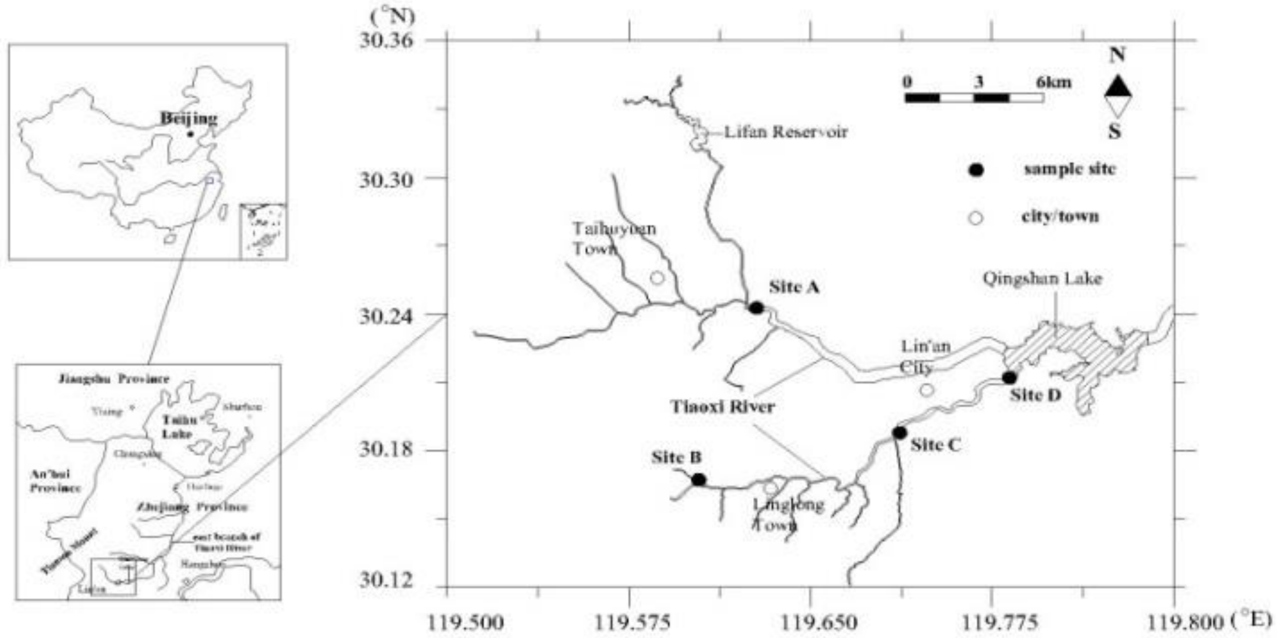

Figure S1 The location of field sites of four bulk sediments in the Tiaoxi River. The map were come from web of "Data Sharing Infrastructure of Earth System Science" http://www.geodata.cn. All of the maps used in the manuscript are free.

Table S1 The distribution of AOA populations from various Chinese inland waters in different AOA species

Sample ID	Inland waters	Number of total sequences	Number of sequences distributed in different AOA species																																	
			I.1b																												I.1a-associated		I.1a			
			1	2	3	4	5	6	7	8	9	10	11	12	13	14	15	16	17	18	19	20	21	22	23	24	25	26	27	28	29	30	31	32	33	34
1	Tarim River	22		1	7				2	1				3													1						2		5	
2	Bosten	20			6				7					2											1			1					3			
3	Tianchi	15		3										6					2						1		3									
4	Aydingkol	19			4					1				2					1					1						2			3		5	
5	Yellow River	23			4					1				4	2									2	1		4			4			1			
6	Yinchuan	29			3	1																					21			4						
7	Ulansuhai	17			3				2					5					1					2	1		2			1						
8	Yongding	11		1	3					1									1					2			1								2	
9	Baiyangdian	33		9	12			3	3					3		3																				
10	Kunming	14			9					4	1																									
11	Yuanmingyuan	35	3		14		1			8				5					1								1						2			
12	Shahe	30			10									15																	5					
13	North Canal	27				4				3				10		2								4			2				1				1	
14	North Canal	34	2	2	8					12				8		2																				
15	Songhua	32												6		12			4		2		6												2	
16	Panjin	10			2									1		5								1			1									
17	Red Beach	12			1				3					2		1						1					3	1								
18	Shangqiu	58		1	6				6					17					2			2				1				4			5	3	11	
19	Chaohu	14							1					3		3																		1	6	
20	Tiaoxi	44			2					2		1		15		7			3						2		7							1	4	
21	Jiaxing (CW)	12							1					4		4													1					1	1	
22	Jiaxing (paddy)	59												27		6	3		2												1	2	7		11	
23	West Lake	9			1									4													1						1		2	
24	Tieshanping	24																				6										18				
25	Donghu	21			2								1	2		1			1	1	2					4				1	1		4		1	
26	Poyang	33			1									3				1															7	9	8	4
27	Dongting	22			6				1					5																2	3		5			
28	Pearl River	50	3		11			1		4				7							2	1					4						9	4	4	

Table S2 Spearman correlation analysis between PNR, archaeal/bacterial *amoA* abundance and environmental variables

		PNR	AOA abundance	AOB abundance	pH	NH_4^+	NO_x^-	TN	TP	TOM	TC	TS
**PNR**	*r*	1.000	0.020	**0.553****	**-0.266****	0.219	**0.285***	0.115	-0.020	0.009	-0.175	-0.259
	p		0.871	0.000	0.038	0.075	0.025	0.393	0.881	0.944	0.315	0.134
	N	69	69	58	61	67	62	57	57	59	35	35
**AOA abundance**	*r*	0.020	1.000	-0.004	**-0.277***	-0.089	**-0.321****	-0.095	**0.286***	0.039	**-0.755****	**-0.748****
	p	0.871		0.971	0.023	0.425	0.004	0.448	0.022	0.751	0.000	0.000
	N	69	87	75	67	83	78	66	64	68	36	36
**AOB abundance**	*r*	**0.553****	-0.004	1.000	-0.193	0.166	**0.497****	**0.529****	0.078	**0.382****	**0.365***	**0.484****
	p	0.000	0.971		0.155	0.160	0.000	0.000	0.567	0.003	0.044	0.006
	N	58	75	75	56	73	68	58	56	58	31	31

Signif. Codes (two tail): 0.01‘**’; 0.05 ‘*’.
TN: Total Nitrogen; TOM: Total Organic Matter; TP: Total Phosphorus; TC: Total Carbon; TS: Total Sulphur

Table S3 Results of multiple linear stepwise regressions on PNR

Results of the model	Adjuster R^2	p-value of the model	p-value of the variables	
PNR = 114.27 + 11.53 (lg AOB) – 20.16 pH	0.212	0.015	Intercept	0.240
			lg AOB	0.050
			pH	0.067

All variables left in the model are significant at the 0.150 level.

Table S4 The aquatic characteristics of samples site in Tiaoxi River

Site	Plant	NH_4^+ (mg L^{-1})	TN (mg L^{-1})	TP (mg L^{-1})	pH
A	*Paspalum Gramineae*	0.1-0.5	0.5-1.2	0.1-0.3	7.2-8.1
B	*Echinochloa Gramineae*	0.1-0.7	0.6-1.5	0.1-0.5	7.2-8.3
C	*Echinochloa Gramineae*	0.2-1	2-3	0.3-0.9	7.4-8.7
D	*Polypogon Gramineae*	1.5 -3	3-6	0.5-1.8	7.4-8.8

Table S5 Spearman correlation coefficients describing the relationships between AOA and AOB abundance and potential nitrification rates in Tiaoxi River (n = 12).

comparisons	Spearman correlation coefficients				
	all	Site A	Site B	Site C	Site D
AOA vs rate	0.252	0.500	0.500	0.573	0.310
AOB vs rate	**0.783****	**1.000****	-	0.812	0.936

Signif. Codes (two tail): 0.01‘**’; 0.05 ‘*’.

Table S6 The physical-chemical parameters of sampled sediments

Inland waters	Type	TOM	NH_4^+	NO_x^-	TN	TC	TS	TP	pH
		g kg^{-1}	mg kg^{-1}	mg kg^{-1}	g kg^{-1}	g kg^{-1}	g kg^{-1}	g kg^{-1}	
Tarim River	River riparian	0.7-5.0	0.8-1.7	0.0-0.2	0.2-0.4	22.8-32.9	0.3-0.4	0.5-0.6	8.4-8.9
Bosten	Lake littoral	13.1-78.1	3.9-26.6	0.8-7.6	0.7-3.2	41.6-63.3	1.6-9.8	0.3-0.6	8.0-8.6
Tianchi	Lake littoral zone	25.5	0.8	0.2	1.5	14.4	0.7	0.8	7.66
Aydingkol	Lake	2.1-12.9	1.5-43.2	4.2-239.0	0.7	12.1	0.7	0.7	7.4-8.5
Yellow River	River riparian	3.9-44.4	0.2-62.8	0.7-6.2	0.3-4.1	16.2-38.6	1.1-2.0	0.5-2.3	8.5-8.5
Yinchuan	Paddyfield soil	-	-	-	-	-	-	-	-
Ulansuhai	Lake	10.7-70.0	14.0-23.6	1.2-3.8	0.7-3.9	21.2-52.7	1.9-11.4	0.6-0.6	8.3-8.6
Yongding	River	75.4-120.6	2.5-9.0	16.1-19.5	3.0-5.7	41.0-117.6	3.3-5.3	0.5-1.0	7.6-7.8
Baiyangdian	Lake ecotone	26.6-74.6	95.6-378.1	7.9-20.4	1.3-4.8	13.0-24.1	0.9-8.7	0.4-1.1	7.6-8.5
Kunming	Lake	-	25.3	0.2	-	-	-	-	-
Yuanmingyuan	Lake	16.5-17.4	6.1-40.0	0.2-0.8	0.9-1.0	17.7-19.1	1.1-1.6	0.5-0.6	8.0-8.1
Shahe	Reservoir	8.4	2.6	2.0	5.3	4.1	0.6	0.1	7.2-8.0
North Canal	Canal	-	-	-	-	-	-	-	-
North Canal	Groundwater	5.8	39.8	9.7	0.9	9.8	1.7	0.08	7.8
Songhua	River riparian	9.6-16.3	9.0-268.3	0.0-16.7	0.7-1.6	7.6-8.6	0.2-0.3	0.1-0.7	7.1-7.9
Panjin	Swamp	10.6-12.3	35.8-49.4	6.6-11.6	1.8-2.0	16.0-21.7	0.5-0.6	0.05-0.06	8.3-8.4
Red Beach	Tidal land	8.0-13.5	10.4-14.2	8.4-13.6	1.4-2.2	3.6-12.5	0.1-0.3	0.03-0.13	8.3-8.7
Shangqiu	Reservoir	17.5-31.4	9.2-15.0	0.6-16.8	0.3-3.3	25.1-27.1	2.0-2.0	0.1-0.7	7.6-8.1
Chaohu	Lake	9.8-14.9	13.9-24.6	0.3-0.8	0.7-1.3	7.1-21.0	0.8-1.2	0.5-1.2	6.6-7.5
Tiaoxi	River	4.9-45.6	7.2-79.3	0.8-11.5	0.8-4.3	9.4-46.0	0.2-3.6	1.2-2.7	7.7-8.7
Jiaxing	Constructed wetland	5.1-42.8	3.3-67.0	1.8-10.4	0.6-3.2	3.4-26.4	0.5-1.8	0.5-6.9	6.8-7.5
Jiaxing	Paddy-field	1.6-44.5	6.7-178.8	1.1-40.6	0.3-3.1	3.4-32.6	0.1-5.8	0.1-0.5	6.6-8.2
West Lake	Lake	-	108.4	2.07	-	-	-	-	-
Tieshanping	River	-	-	-	-	-	-	-	3.9-4.0
Donghu	Lake littoral	8.3-119.7	0.2-0.9	0.6-11.2	0.8-1.8	6.5-35.1	0.4-13.8	0.7-1.1	7.9-8.3
Poyang	Lake	9.4-17.4	19.6-43.2	0.0-1.2	0.3-1.2	7.0-11.6	0.3-0.4	0.4-0.5	6.9-7.4
Dongting	Lake littoral zone	6.9-31.6	0.4-3.5	0.0-11.0	0.5-3.5	9.1	0.4	0.5	8.1
Pearl River	Estuary	7.4-64.6	24.0-68.7	1.2-16.8	0.5-5.0	10.4-43.6	1.6-5.7	1.0-9.6	7.1-7.8

Table S7 Primers and conditions for qualitative/quantitative PCR

Specificity	Primer	Sequence (5'-3')	Thermal profiles	Reference
AOA	Archaea-*amoA*F (PCR)	STAATGGTCTGGCTTAGACG	95 ℃ 5min; (94 ℃ 45s, 53 ℃ 60s, 72 ℃ 60s) for 30 cycles; 75 ℃ 15min	23
	Archaea-*amoA*R (PCR)	GCGGCCATCCATCTGTATGT		
AOA	Archaea-*amoA*F (qPCR)	STAATGGTCTGGCTTAGACG	50 ℃ 2min, 95 ℃ 30s; (95 ℃ 10s, 53 ℃ 30s, 72 ℃ 60s) for 40 cycles; 72 ℃ 1min	4, 23
	Archaea-*amoA*R (qPCR)	GCGGCCATCCATCTGTATGT		
AOB	*amoA*-1F (qPCR)	GGGGTTTCTACTGGTGGT	50 ℃ 2min, 95 ℃ 30s; (95 ℃ 10s, 55 ℃ 30s, 72 ℃ 60s) for 40 cycles; 72 ℃ 1min	26, 4
	amoA-2R (qPCR)	CCCCTCKGSAAAGCCTTCTTC		
